# Long-term effects of a prehospital telemedicine system on structural and process quality indicators of an emergency medical service

**DOI:** 10.1038/s41598-023-50924-5

**Published:** 2024-01-03

**Authors:** Hanna Schröder, Stefan K. Beckers, Christina Borgs, Anja Sommer, Rolf Rossaint, Linda Grüßer, Marc Felzen

**Affiliations:** 1 Department of Anesthesiology, Medical Faculty RWTH Aachen University, University Hospital RWTH Aachen, Pauwelsstrasse 30, 52074 Aachen, Germany; 2 Aachen Institute for Rescue Management and Public Safety, City of Aachen and University Hospital RWTH Aachen, Pauwelsstrasse 30, 52074 Aachen, Germany; 3 Medical Direction of Aachen Fire Department, Stolberger Strasse 155, 52068 Aachen, Germany

**Keywords:** Preclinical research, Health care

## Abstract

The benefits of a telemedical support system for prehospital emergency medical services include high-level emergency medical support at the push of a button: delegation of drug administration, diagnostic assistance, initiation of therapeutic measures, or choice of hospital destination. At various European EMS sites telemedical routine systems are shortly before implementation. The aim of this study was to investigate the long-term effects of implementing a tele-EMS system on the structural and procedural quality indicators and therefore performance of an entire EMS system. This retrospective study included all EMS missions in Aachen city between 2015 and 2021. Regarding structural indicators of the EMS system, we investigated the overall number of emergency missions with tele-EMS and onsite EMS physicians. Furthermore, we analyzed the distribution of tracer diagnosis and process quality with respect to the time spans on the scene, time until teleconsultation, duration of teleconsultation, prehospital engagement time, and number of simultaneous teleconsultations. During the 7-year study period, 229,384 EMS missions were completed. From 2015 to 2021, the total number of EMS missions increased by 8.5%. A tele-EMS physician was consulted on 23,172 (10.1%) missions. The proportion of telemedicine missions increased from 8.6% in 2015 to 12.9% in 2021. Teleconsultations for missions with tracer diagnoses decreased during from 43.7% to 30.7%, and the proportion of non-tracer diagnoses increased from 56.3% to 69.3%. The call duration for teleconsultation decreased from 12.07 min in 2015 to 9.42 min in 2021. For every fourth mission, one or more simultaneous teleconsultations were conducted by the tele-EMS physician on duty. The implementation and routine use of a tele-EMS system increased the availability of onsite EMS physicians and enabled immediate onsite support for paramedics. Parallel teleconsultations, reduction in call duration, and increase in ambulatory onsite treatments over the years demonstrate the increasing experience of paramedics and tele-EMS physicians with the system in place. A prehospital tele-EMS system is important for mitigating the current challenges in the prehospital emergency care sector.

## Introduction

Over the past decade, telemedical applications have improved patient care at multiple levels^1^. At various emergency medical service (EMS) sites in Germany, routine systems are in place or shortly before implementation to support paramedics in prehospital emergency missions through teleconsultation with a telemedical emergency physician (tele-EMS physician)^2–4^. Aachen city, Germany implemented a prehospital telemedicine system in 2014 and has since applied telemedical care for prehospital emergency patients in daily routines^5^. Details on the system are elaborated within the “Method” section.

Several regional studies, and data from additional implementation sites in Germany (e.g., Greifswald, Goslar, and Bavaria), have demonstrated structural benefits of EMS and the therapeutic advantages resulting from prehospital teleconsultation^3,6,7^. Overall, several publications have investigated the tele-EMS system regarding the allocation of resources, professional education of tele-EMS staff, reasonable reduction of the involvement of onsite emergency physicians (onsite EMS physicians), and investigation of documentation quality or time of care for various tracer diagnoses^8–10^. Additionally, technical infrastructure, performance, usability, and acceptance are described in detail in the literature^5,11,12^. In the field of patient care research, tele-EMS physicians provide care for stroke, hypertensive crisis, acute coronary syndrome, analgesia, immobilization in mild trauma, diagnostic concordance of tele-EMS physicians, and life-threatening missions^10,13–17^. Furthermore, one randomized controlled trial has reported non-inferiority of tele-EMS physicians compared to onsite EMS physicians lately^18^.

However, these studies did not assess the long-term effects of implementing a tele-EMS system from a healthcare system perspective. Moreover, an evaluation of the long-term impact of a telemedical system on the quality and performance of EMS is missing. Considering that the German EMS is traditionally a physician–staff system, only a few studies can be found on quality indicators and quality assurance in this field^19,20^. To our knowledge, no study has investigated long-term quality indicators in a prehospital EMS system that implements tele-EMS physician-based support. Key stakeholders, including EMS management, paramedics, emergency physicians, and training supervisors, should consider long-term effects before implementation.

We aimed to determine the long-term effects of the availability of a holistic telemedical system in EMS by analyzing system-wide changes in the structural and process indicators of the telemedical system.

## Methods

This retrospective observational study was initiated and performed at the Aachen Institute for Rescue Management and Public Safety, in close cooperation with the Department of Anesthesiology, RWTH Aachen University, Germany. The study protocol has not been published a priori.

### Setting of tele-EMS system & qualification of staff

The telemedicine system applies wide-ranging technical solutions to provide as much helpful information from the patient and the ambulance team on scene.

It consists of two main components: a teleconsultation center managed by an experienced emergency physician (tele-EMS physician) and ambulances equipped to enable teleconsultations for paramedics from any scene. The technical equipment of every ambulance includes an in-car communication unit as well as a mobile communication unit connected to a defibrillator monitor (corpuls^3^; GS Elektromedizinische Geräte G. Stemple, Kaufering, Germany). It allows for audio data transmission, real-time vital data transmission (including heart rate, blood pressure, oxygen saturation), real-time 6- and 12-leads-ECG, picture transmission from a smartphone as well as video streaming inside and outside the ambulance. There is a printing option for teleconsultation reports inside the ambulance.

The teleconsultation center is provided with a workplace similar to a dispatch center, where the tele-EMS physician handles an integrated software solution “TM-Doku”, (*Telemedical documentation*™; by umlaut telehealthcare, Aachen, Germany) that was specifically developed for this purpose and continuously refined since 2014. The software enables mission documentation, video and audio-conferencing system, visualization of patient vital data as well as GPS location of ambulances and hospitals of the region. Additionally, all data from corpuls^3^ equipped and connected ambulances is assessable via the corpuls web application and allows a redundancy for vital signs, patient data and measurement functions for 12-leads-ECG. Therefore, this wide-ranging technical composition differentiates from telemedical solutions that basically apply call-back opportunities for teams on scene. Several earlier publications on the technical performance of the system have reported on the details of the technical concept and further explain on transmission quality and redirection of data traffic via a local wireless network^4,5^.

In North Rhine-Westphalia, the qualification requirements for tele-EMS physicians are regulated by the regional medical council and include at least 500 onsite emergency operations as an EMS physician, board certification in an emergency-related medical specialty (e.g., anesthesiology, surgery, or internal medicine), and course certificates for intensive care transport. These qualifications permit staff to complete an additional 28-h curriculum in tele-emergency medicine^21^. Local EMS regulations may require additional qualifications or further assessments. In Aachen, tele-EMS physicians undergo supervised training in a teleconsultation center for a week before taking responsibility for their missions. For paramedics, a one-day technical instruction course and a consultation training course were conducted to train them in handling technical equipment in the ambulance and to teach them how to follow the structured teleconsultation sequence.

### Data sources, eligibility criteria

The software, *Telemedical documentation*, displays all transmitted data during teleconsultation and is operated by the tele-EMS physician. All diagnostic measures, values, and therapeutic interventions were documented using standardized emergency protocols and stored electronically. All items of the standardized protocols were exported into a database format for Microsoft Excel and were available for analysis.

Furthermore, data-sets of EMS missions in Aachen from 01.01.2015 to 31.12.2021 were exported from the computer-aided dispatch system of the fire department in Aachen city and matched with the protocol data. The dataset from the dispatch system included timestamps of the emergency call, time of dispatch, deployment of EMS resources, arrival at the scene, transport to the hospital, and arrival at the hospital.

Tele-EMS rollout started in spring 2014 and by December 2014, all ambulances in Aachen city were given essential equipment. Therefore, the study period was set from 01.01.2015 to 31.12.2021, spanning seven years. All analyses were conducted in accordance to relevant guidelines and regulations.

### Study outcomes

To evaluate the long-term effects of the operation of a telemedical system over seven years on the EMS system, the following indicators were investigated:

1. structural indicators of the EMS system
2. distribution of EMS-tracer and non-EMS-tracer diagnoses of emergency medical patients
3. indicators for process quality of teleconsultations

Concerning structural indicators, we investigated: (a) overall number of emergency missions, (b) progression of ratio of missions with tele-EMS and onsite EMS physicians out of all missions, and (c) telemedical operations with an additional need to call for an onsite EMS physician (here the onsite EMS physician was notified secondarily by the tele-EMS physician or ambulance at the scene, in the following referred to as a 'secondary alarm').

Regarding the distribution of tracer and non-tracer diagnoses, frequencies were analyzed through time to determine the scenarios for which paramedics conduct teleconsultations. Generally, tracer diagnoses represent certain serious illnesses and injuries that require preclinical and clinical care by trained staff using specific technical resources within a certain timeframe in accordance with the recommendations of medical associations and guidelines^22^. Historically, the term tracer diagnoses was associated with physician-staffed EMS indications, we explicitly do relate to EMS indications for paramedic staffed ambulances here. Clearly structured treatment pathways for prehospital tracer diagnoses exist for handling prehospital emergency cases in Germany^23^. In Aachen’s dispatch center the dispatcher handling the emergency call has opportunity to decide whether to send a paramedic-staffed ambulance solely (with opportunity for teleconsultation) or to also alarm the physician-staffed vehicle for severe cases (e.g. severe dyspnea, hemodynamic instability, unconsciousness, resuscitation, major trauma etc.). In this study tracer diagnoses were only analyzed for EMS missions in which paramedics conducted a teleconsultation.

We categorized document-related diagnoses into clusters of tracer and non-tracer diagnoses. Clusters were assigned following established standard operating procedures (SOPs) for paramedics according to the North Rhein-Westphalia compendium for EMS from 2023 and joint SOPs for EMS staff from the medical EMS directors of Baden-Württemberg, Mecklenburg-Vorpommern, North Rhine-Westphalia, Sachsen, and Sachsen-Anhalt from 2021^24,25^. The clusters of tracer and non-tracer diagnoses are shown in Table 1.

**Table 1:** Cluster for tracer and non-tracer diagnoses (left column) and assigned diagnoses (right column).

Cluster of diagnoses	Included major diagnoses
Tracer	
Analgesia total	Trauma extremity, abdominal emergencies, back pain (including lumbar pain)
Acute coronary syndrome (ACS)	Angina pectoris, non-ST-elevation myocardial infarction, ST-elevation myocardial infarction
Arrhythmia	Bradycardia, tachycardia
Bronchial obstruction	Chronic obstructive lung disease (COPD), asthma
Hypertensive emergency	
Other tracer diagnoses	Anaphylaxis, acute aortic syndrome, aspiration, cardiac decompensation, hypo-/hyperglycemia, intoxication, major trauma, pulmonary embolism, resuscitation, thermic and electrical injury,
Seizure	Convulsion with and without fever
Sepsis	Pneumonia, urosepsis, sepsis of unclear focus
Stroke	
Non-tracer	
Syncope	Temporary loss of consciousness of any cause but a tracer diagnoses (ACS, arrhythmia, seizure, stroke, pulmonary embolism, major trauma,
Psychiatric emergency	Dementia, agitation, depression, suicidality
Respiratory infection	Any diagnose of airway infection (e.g. sinusitis) but not pneumonia and COPD
Neurological emergency	Any other but stroke (peripheral neurologic disorders, neuropathy
Gastro-intestinal infection	Diarrhea with and without fever, emesis, vomiting, nausea
Other non-tracer diagnoses	The cluster contained all cases without available SOP that could not be categorized into any former described cluster. As reasons for consultation and suspected diagnoses diversify very much in this group (> 40), it was not reasonable to list every possible diagnoses

Tracer diagnoses were defined by availability of established standard operating procedures (SOPs) for paramedics (method section) according to North Rhein-Westphalia compendium for emergency medical service (EMS) from 2023 and joint standard operation procedures for EMS from medical EMS directors of Baden-Württemberg, Mecklenburg-Vorpommern, North Rhine-Westphalia, Sachsen, Sachsen-Anhalt from 2021. (25, 26).

Concerning indicators for the process quality of teleconsultations, we investigated (a) time until first teleconsultation after ambulance dispatch, (b) duration of actual teleconsultation call between paramedics and tele-EMS physicians, (c) ambulance time on scene, (d) proportion of ambulatory treatments on scene (onsite patient treatment without transportation to a hospital), and (e) progression of simultaneous teleconsultations for the EMS physician. A simultaneous consultation was defined as an additional teleconsultation call that occurred when a previous teleconsultation was still running. Even if only one teleconsultation can be active on the phone, several other teleconsultations can run in the system, displaying vital signs and sending information such as ECG and pictures. This requires the tele-EMS physician to prioritize and switch between missions.

Analysis of time indicators was applied to all tele-EMS missions conducted by an Aachen ambulance, and operating data were available from the computer-aided dispatch system of the corresponding center. These indicators were chosen to identify process changes over time and develop hypothesis regarding the learning effects of users and adaptation mechanisms.

### Statistical analysis

Plausibility checks of protocol data were conducted to reduce and correct obvious mistakes in documentation before statistical analysis. Descriptive analyses were conducted using Microsoft Excel for Office365 (Microsoft Corporation, Washington, USA). Categorical variables were presented as numbers (%). Fisher’s exact test was used to compare the proportions and rates between 2015 and 2021. Unpaired t-test was used to compare continuous variables. *P*-values < 0.05 were considered significant.

No exclusion criteria were applied to the dataset during the study period. Therefore, a selection bias did not influence the analysis. Missing values can naturally occur in analyzing medical records of onsite EMS physicians as well as in tele-EMS documentation.

### Ethics approval and consent to participate

The Ethics Committee at the RWTH Aachen Faculty of Medicine (Pauwelsstraße 30, 52,074 Aachen, Germany) approved the analysis without any constraints (approval number:109/15). The need for informed consent was waived by the Ethics Committee at the RWTH Aachen Faculty of Medicine (Pauwelsstraße 30, 52074 Aachen, Germany, Head: Prof. Hausmann), the Center for Translational and Clinical Research (CTC-A) of RTWH Aachen University, and the responsible data protection officers, because this retrospective analysis was performed anonymously in the context of legally required quality assurance under the responsibility of municipal authorities.

## Results

During the study period, 229,384 emergency missions occurred in Aachen city and were attended by at least one ambulance. A tele-EMS physician was consulted by the ambulance team for 23,172 (10.1%) missions.

### Structural indicators of Aachen EMS system

**Figure 1:**
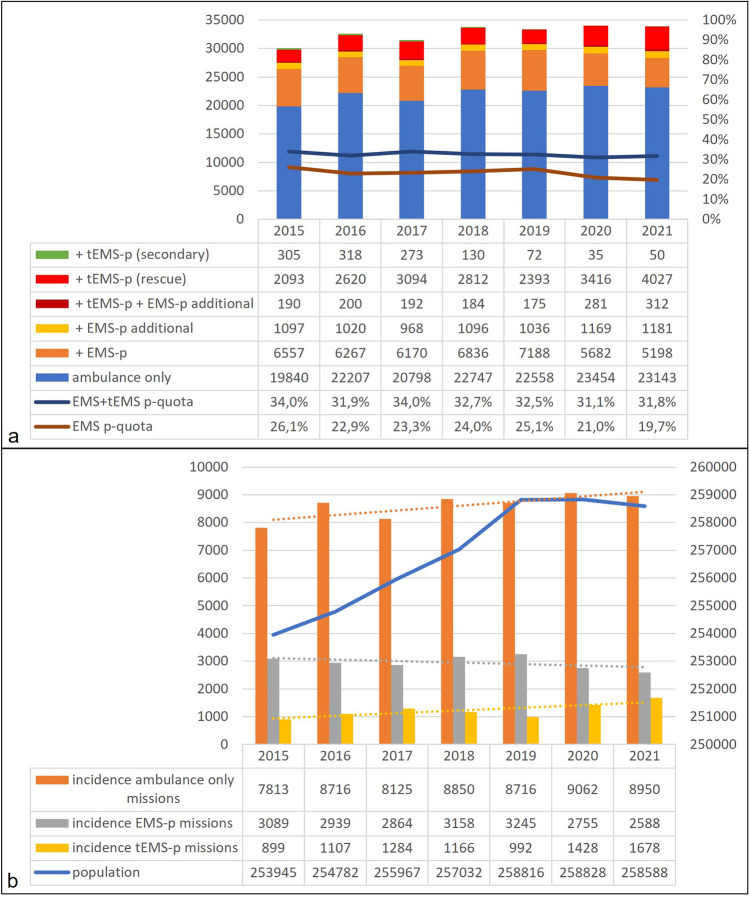
(**a**) EMS missions in Aachen city from 2015 to 2021. Left axis shows absolute numbers of missions, right axis shows percentage out of all rescue missions. + tEMS-p (secondary) = tele-EMS physician accompanied ambulance during a secondary/interhospital transport; + tEMS-p (rescue) = ambulance conducted teleconsultation with tele-EMS physician in a rescue mission; + tEMS-p + EMS-p additional = tele-EMS physician called for additional onsite EMS physician (secondary alarm); + EMS-p additional = ambulanced called for additional onsite EMS physician (secondary alarm); + EMS-p = ambulance and onsite EMS physician; ambulance only = no physician involved; EMS + tEMS p-quota = proportion of rescue mission where a physician is involved EMS p-quota = proportion of rescue missions where an onsite EMS physician is involved. (**b**) Incidences of EMS missions depending on involved resources. Incidences represent missions per 100.000 per year for ambulance only, involved EMS physician (EMS-p) and involved tele-EMS physician (tEMS-p). Population represents inhabitants of the city of Aachen from the statistical yearbook.

Figure 1a presents the number of overall missions, progression of the ratio of missions with tele-EMS and onsite EMS physicians out of all missions, and operations with additional demand from an onsite EMS physician in Aachen city over seven 7. In general, the number of EMS missions increased continuously by 8.5% between 2015 and 2021.

This increase was reflected in the rising proportion of ambulance missions without physician support, from 67.0% to 71.1% (*p* < 0.001). The proportion of telemedical missions increased from 8.6% to 12.9% (*p* < 0.001). For onsite EMS physician missions, the proportion decreased from 26.1% to 19.7% (*p* < 0.001), this reflects a reduction of 32,5%. Over the years, the proportion of secondary alarms of onsite EMS physicians during teleconsultation remained unchanged (7.3% in 2015 vs. 7.1% in 2021 (*p* = 0.7371]), and the overall number of secondary alarms (including those without teleconsultation) increased from 16.4 to 22.3% (*p* < 0.001). The incidences of missions per 100,000 inhabitants for the different EMS resources are presented in Fig. 1b.

**Figure 2:**
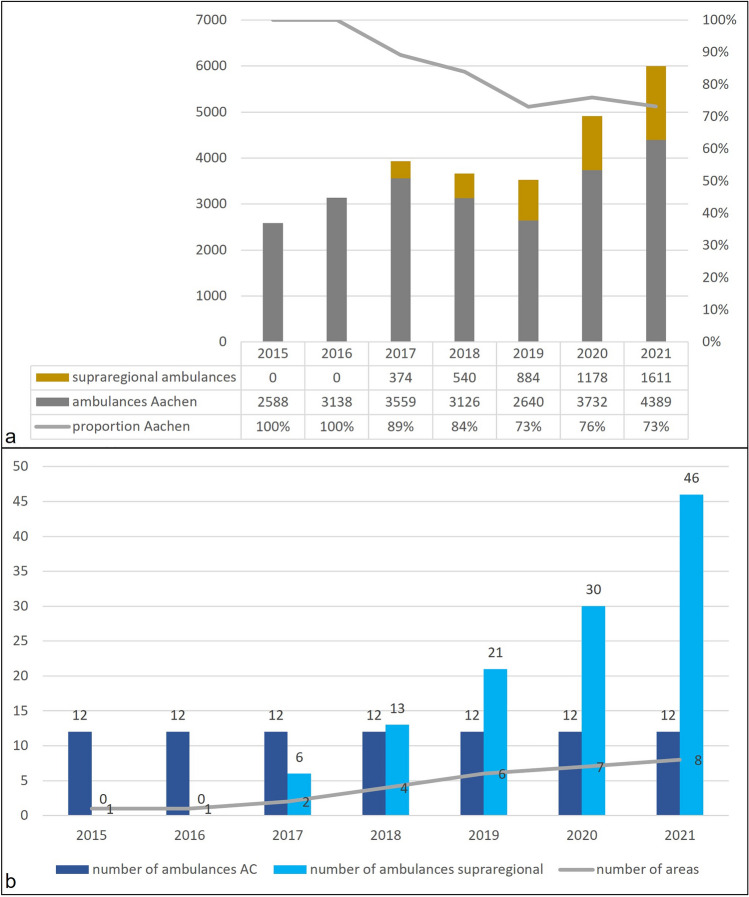
(**a**) Telemedical missions in the tele-EMS center of Aachen including supraregional ambulances. Ambulances Aachen = all teleconsultations with ambulances from EMS of Aachen (equal to the sum of cases in Fig. 1 of tEMS-p (secondary) + tEMS-p (rescue) + tEMS-p + EMS-p additional). (**b**) Number of ambulances and EMS areas connected to the tele-EMS center Aachen over the years. AC = city of Aachen.

Independent of developing rescue missions in Aachen city, tele-EMS physicians began supporting ambulances from other EMS regions in 2017. (These are intentionally not included in Fig. 1 because they do not belong to registered Aachen EMS missions). Teleconsultations significantly increased yearly (Fig. 2a) while supra-regional ambulances (outside Aachen city) were increasingly connected to the Aachen tele-EMS center (Fig. 2b).

### Distribution of tracer and non-tracer diagnosis in tele-emergency missions

Here, we excluded secondary and interhospital transport (n = 1183) from the total number of tele-EMS missions (n = 23,172), as these were not considered initial rescue missions with an acute need for emergency medical intervention. Thus, the major diagnoses of 21,989 teleconsultations from rescue missions were analyzed.

**Figure 3:**
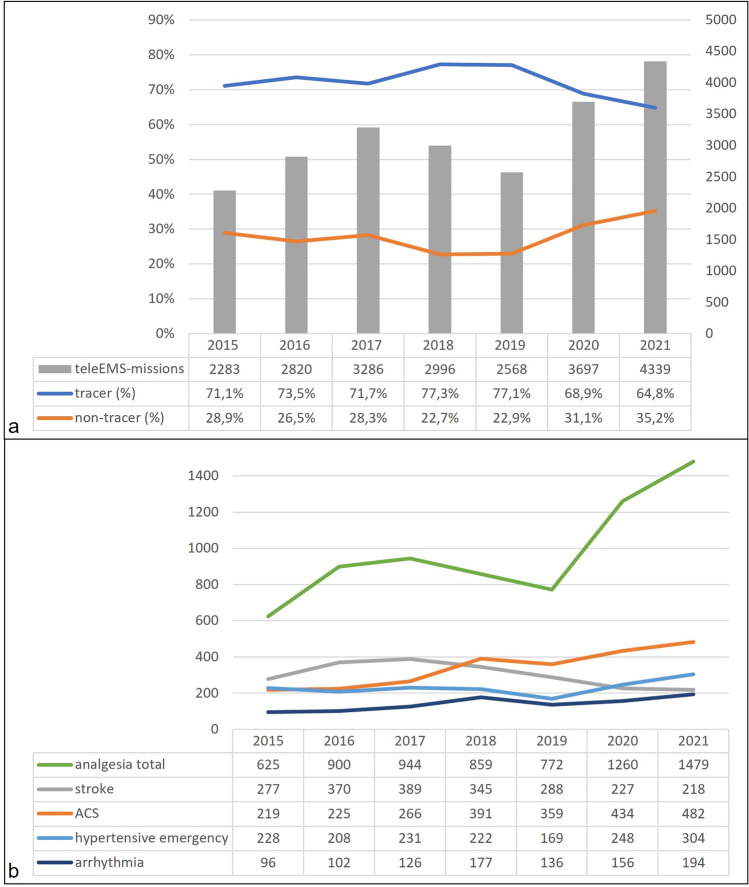
(**a**) Numbers of teleconsultations for tracer and non-tracer diagnoses in rescue mission in Aachen city from 2015 to 2021. (**b**) Distribution and progression of the major five tracer diagnoses.

In 2015, ambulances conducted teleconsultations for tracer diagnoses in 71.1% (n = 2283) of rescue missions. By 2021, this proportion declined significantly to 64,8% (n = 4339; *p* < 0.001), although the overall number of missions increased (Fig. 3a). For non-tracer diagnoses, this development showed the opposite effect, increasing from 28.9% to 35.2% (*p* < 0.001).

The most commonly documented diagnoses were analgesia 31% (for trauma of an extremity 15.6%, abdominal emergency 11.6%, lumbar pain 4.0%), acute coronary syndrome (ACS) 10.8%, stroke 9.6%, hypertensive emergency 7.3% and arrhythmia 4.5%. These groups represent tracer diagnoses using available SOPs. The progression of their numbers over seven years is presented in Fig. 3b. The absolute numbers of the most frequent diagnoses (including tracer and non-tracer diagnoses) during the seven-year study period are available in the additional material (see supplementary file 1).

### Indicators for process quality of teleconsultations

**Figure 4:**
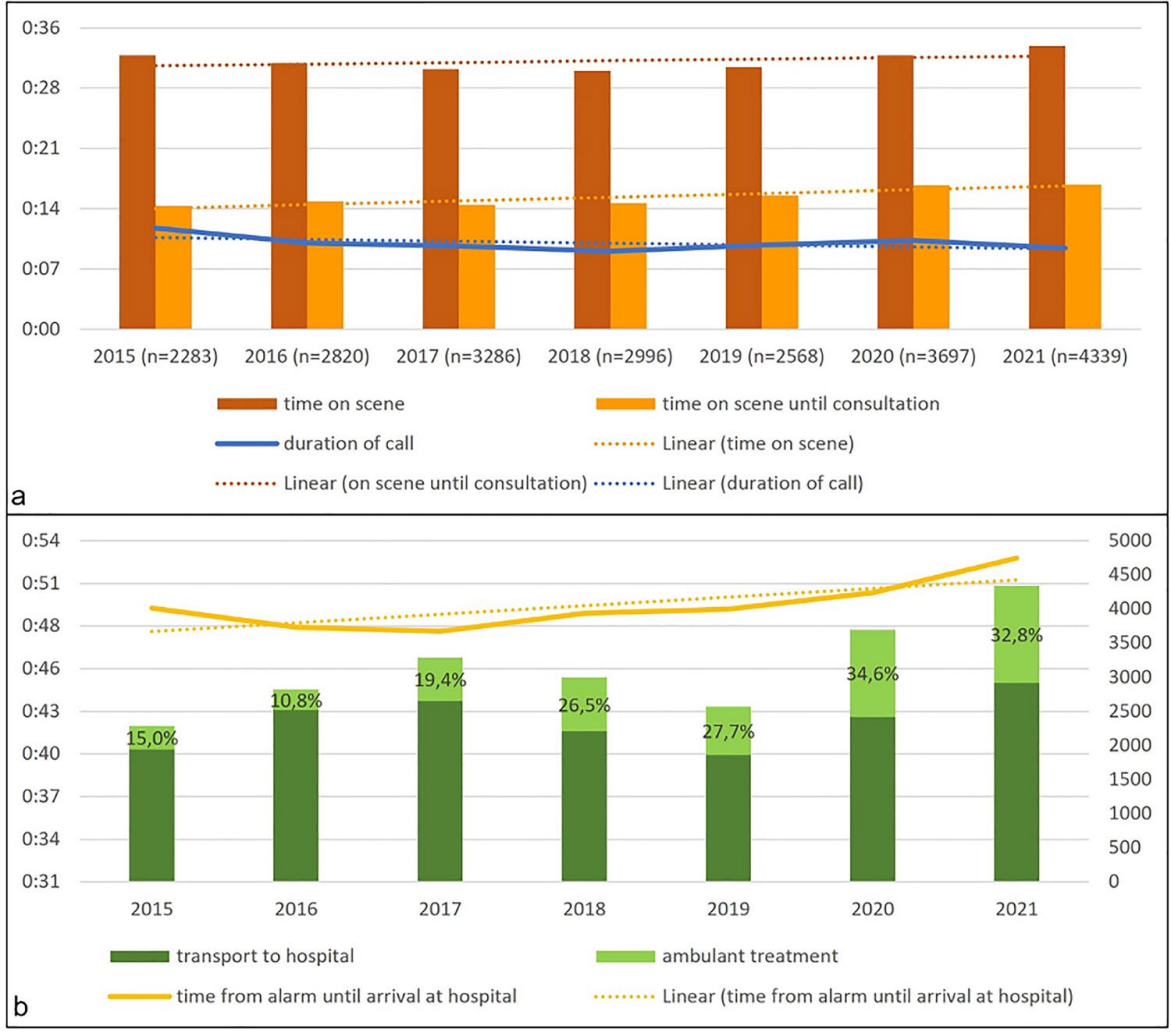
(**a**) Time on scene, time until teleconsultation and duration of call. (**b**) Proportion transport to hospital, ambulatory treatment and prehospital time for emergency patients in tele-EMS missions (from alarm until arrival at hospital). All values are presented in minutes.

The mean latency from ambulance arrival to the call for teleconsultation was 14.44 min in 2015. This time period increased significantly over the years up to 17.17 min (*p* < 0.001) (Fig. 4a). The mean duration for a teleconsultation call started off with 12.07 min in 2015 and decreased to 9.42 min in 2021 (*p* < 0.001) (Fig. 4a). The mean overall time that an ambulance in Aachen city was occupied with a mission including teleconsultation (from alarm until arrival at the hospital) was 50.11 min. It slightly (non-significantly) increased to 53.33 min in 2021 (Fig. 4b). The proportion of ambulatory treatments at the scene (patient treatment without transport to a hospital) more than doubled in seven years from 15.0% (n = 342) in 2015 to 32.8% (n = 1424) in 2021 (*p* < 0.001).

**Figure 5:**
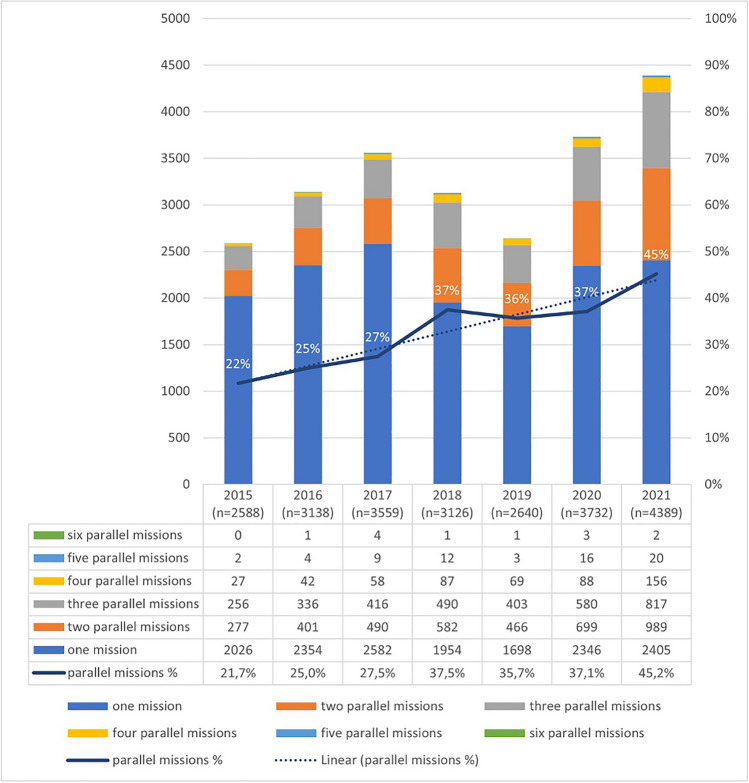
Parallel missions in the tele-EMS center (ambulances from Aachen & supraregional ambulances). Right axis represents proportion of missions in %; left axis represents absolute numbers of teleconsultations; the line of parallel missions and numbers in blue bar represent relative frequency of occurrence of one or more parallel mission when a mission is already running in percentage (white numbers).

The number of simultaneous teleconsultations handled by tele-EMS centers increased over the past seven years, as shown in Fig. 5.

The maximum number of teleconsultations handled simultaneously was six. During the study period, the overall occurrence of one or more parallel teleconsultations increased from 21.7% (n = 2588) in 2015 to 45.2% (n = 4389) in 2021. There were 58 ambulances connected to the tele-EMS system in 2021. Of all parallel teleconsultations (n = 1984), two took place in 49.5% (n = 989), three in 41.2% (n = 817), four in 7.9% (n = 156), five in 1.0%, and a maximum of six parallel teleconsultations in 0.1% (n = 2) (Fig. 5).

## Discussion

This first-time analysis of telemedical emergency care in an EMS over seven years aimed to elucidate the factors influencing such a system. The major findings revealed increasing proportions of teleconsultations, decreasing proportions of onsite EMS physician rescue missions, rising simultaneous missions, shortening call durations, and increasing proportion of ambulatory treatments over the years.

### Development of mission numbers in the EMS system of Aachen

As reported previously, onsite physician ratio was 36% in the year before implementation of the telemedical system^8^. During the study period the ratio decreased from 26.1% in 2015 to 19.7% in 2021. Therefore, it seems comprehensible that the ratio of missions with tele-EMS participation increased from 7.9% to 12,1%. Even taking into account the local population developments and increase of incidence per 100,000 of overall EMS missions, Fig. 1b reveals declining incidence for onsite physicians compared to increasing incidence of tele-EMS missions. These patients received medical treatment from a remote physician while sparing the onsite EMS physician from life-threatening missions requiring physical presence and manual skills.

Strikingly, there is a recognizable peak of EMS physician missions in 2018 and 2019 and a drop of tele-EMS missions in 2019. Most likely this is related to the conduction of the randomized, controlled, parallel-group trial “TEMS” (“Telemedical support for prehospital Emergency Medical Service”, NCT02617875), which was the first RCT in this field to compare non-inferiority of patient treatment of a tele-EMS physician versus an onsite physician in 1500 missions^18,26^. The study design of the TEMS trial required a randomized alarm from an onsite physician for a potential tele-EMS indication from August 2018 until September 2019. Therefore, ambulance teams conducted fewer teleconsultations and onsite EMS physicians’ missions increased during this period.

In relation to other implemented telemedical systems in EMS there is only few pilot data available that does not include comparable tele-EMS quotas or incidences. First data from the telemedical system of Goslar hast reported 1968 tele-EMS missions over a year in a region of 137,000 inhabitants and 14 connected ambulances^3^. Former data from the City of Greifswald reports a reduction of onsite EMS physician missions about 7% after implementation of the telemedical EMS system^7^. Overall the very limited comparability of available data calls for standardized quality indicators in this field.

Recently, the University of Maastricht analyzed the dimensions of tele-EMS systems in North Rhine-Westphalia and proposed a tele-EMS system for approximately 1.5 million inhabitants^27^. Therefore, the number of ambulances connected to the tele-EMS system does not seem to be the appropriate measure for scaling such a system because the occupancy/number of missions may differ depending on the geographical position of the ambulance [e.g., rural vs. urban areas]^27^. When analyzing the development of teleconsultations from the perspective of the tele-EMS center, the number of teleconsultations from external ambulances outside Aachen city gradually increased over the years as more supra-regional ambulances were connected to Aachen’s tele-EMS, while the number of available 24/7-ambulances in Aachen remained constant with 12 vehicles (Fig. 2b). Figure 2a and b depict this relation between local and supra-regional consultations in context of connected vehicles. We showed that the proportion of calls from Aachen ambulances decreased over the years, although the absolute number of teleconsultations increased, especially in 2020 and 2021. In Aachen, the tele-EMS physician could handle this growth without requiring additional staff resource in the teleconsultation center. Comparative data is not available at present. To sufficiently answer the question of how many tele-EMS physicians are needed for a defined catchment area, this multifactorial environment requires future multicenter comparative research approaches looking at the interaction of number of inhabitants, connected ambulances, and capacity analyses of tele-EMS centers.

### Distribution of tracer diagnosis in tele-emergency missions

Especially in the early years of implementing the tele-EMS system, the SOPs for paramedics also included conditions requiring consultation with the tele-EMS physician [e.g., ECG analysis in ACS, application of opioid analgesics, or drug therapy for arrhythmias]. This clearly explains the frequency distribution of teleconsultation services used for tracer diagnosis. For acute stroke the benefit of hospital prenotification before arrival of the patient promised improvement in the chain of care for stroke patients and therefore was the major intention of conducting teleconsultations for stroke patients^16,28^. Although paramedics are trained to conduct valuable stroke assessment, current research delivers evidence that telestroke evaluation has a greater diagnostic accuracy compared to paramedics in distinguishing hyperacute stroke patients^29^. It needs to be acknowledged that this RCT analyzed teleconsultations conducted by specialized neurologists, and up to today no studies have compared teleconsultations by neurologists versus emergency physicians.

Nonetheless the group of non-tracer diagnoses have also increased over the years. This may be due to the absence of a certain tracer diagnosis (or leading symptom) leading paramedics to ask for tele-EMS physician support to enter the right algorithm-based therapy pathways. The need for physician support in these cases could be easily covered via teleconsultation without an onsite EMS physician. Over the years, with increasing compliance with the system, the reasons for conducting teleconsultations have increasingly diversified. Since 2022 paramedics in Aachen were authorized to perform certain SOP-based therapeutic measures (including drug administration) defined by article §2c of the education law for emergency paramedics in Germany (NotSanG §2c) independently. Therefore, we expected a change in the distribution of major teleconsultation diagnoses in the future.

Another challenge is the continuous tracking of diagnoses in telemedicine systems. This may require the definition of diagnoses for quality surveillance at an early stage of system implementation and allocation of staff resources for. This, together with the challenge of categorizing certain leading symptoms or diagnoses beyond clearly defined tracer diagnoses (and their inconsistent documentation), led to cluster categorization in this study.

### Process indicators of telemedical consultations

With years of system availability, the time until first consultation on a mission has increased. This finding can potentially be explained by the increasing experience of ambulance teams who are trained to complete their diagnostic measures (such as the ABCDE approach, vital signs, ECG, and anamnestic interrogation of the patient) before consulting the tele-EMS physician to process a smooth and quick call. Therefore, there is less need for additional questioning by tele-EMS physicians, and therefore significantly declining duration of calls over the years. This hypothesis would be in line with tele-EMS experiences over the years. But even if the growing routine in using and handling technical systems may support this interpretation, literature mainly recognizes digital skills shortages among health professionals^30^. Nevertheless, there is no specific comparable literature available in this field of process indicators for use of telemedical applications by EMS staff and future research is needed.

Thus, we found increased ambulatory contacts without hospital transport for teleconsultations over the years. As this finding specifically relates to the tele-EMS missions presented in Fig. 4b (and not to the overall EMS missions) the underlying causes can be suspected to be related with the tele-EMS physicians’ medical decisions. One suitable hypothesis would be, that tele-EMS physicians became more comfortable over the years in guiding patients into the ambulatory sector by recommending visits of their family doctor, or the consultation of the medical on-call service during closing hours, when hospital transport in non-urgent bagatelle cases did not appear to be necessary. Former studies have identified age and the location of mission (especially care facilities) as influencing factors for ambulatory contacts^31^. Schehadat et al.^32^ have investigated economic loss from so called OFF-missions (treatment on-site without transport) and a systematic review has identified the early warning scores NEWS/NEWS2 as most suitable for prehospital emergency setting to predict patient deterioration^32,33^. Related to the tele-EMS physicians’ decision making on need for transportation the application of early warning scores appears to be reasonable, but anyhow further research is needed in this field.

Regarding simultaneous tele-EMS missions, until now, it remains unclear how many missions one tele-EMS can actually handle. This is the first attempt to investigate the simultaneous missions in a tele-EMS system. Our data showed that it is likely that two or more teleconsultations run simultaneously, requiring the tele-EMS physician to prioritize and triage between different calls. The highest number of parallel teleconsultations recorded was six missions in only a few cases. In prehospital EMS staff usually has to cope with one single patient at a time, apart from mass casualties. As emergency departments have demanded new educational strategies to train complex multipatient work environments, new SIM-techniques as multiple encounter simulations have been proposed years ago^34^. As mentioned earlier, the occupancy level in a tele-EMS physician depends not only on the number of connected ambulances but also on the duties and spectra of medical cases for the tele-EMS physician. Notably, at the Aachen tele-EMS center, physicians are also responsible for resource management of secondary transports in Aachen city and external EMS regions^35^. The tele-EMS center appears to be such a multipatient work environment, where multitasking, situational awareness and especially effective communication are worth to train tele-EMS physicians for^35^.

Our data confirm the previously described general trend of a continuous increase in the incidence of rescue missions. The implementation of tele-EMS systems in Germany was already recommended by the Federal Joint Committee in 2020 and probably could further reduce unnecessary missions for onsite EMS physicians^36^. It has the potential to reduce but not solve all the challenges faced by the prehospital healthcare sector in Germany. The portion of non-urgent bagatelle cases in EMS and the variety of request for help from the general public will need multi-approach solutions in the future to avoid unnecessary rescue missions, strengthen health education in the population, link emergency and non-emergency ambulatory health care sectors, and provide the right care at the right time to patients. Adjusting the prehospital care system with various approaches as improved emergency call systems, trans sectoral health care provision or community nurses could be parts of the solution^37^. In September 2023 the latest statement of the German governmental commission on the necessary rescue service reform emphasized several recommendations as adaptation of legal regulations, promotion of health literacy, improved patient flow management, as well as data-based quality assurance throughout participation in missions’ registries^38^. Obviously this requires digital networks between healthcare delivering system as well as technically standardized digital solutions for the entire EMS. Currently digital interoperability still poses a great challenge even in implementation of telemedical systems.

## Limitations and future research needs

EMS presents complex systems that differ depending on several general conditions, such as staff education, medical and technical equipment, organizational matters, and emergency call taking and dispatching. Therefore, the scientific interpretation of our results and conclusions should incorporate local practices and contexts. Nevertheless, system changes usually take place over long periods and, especially in the health sector, new forms of care initially struggle with user compliance, technical challenges, and continuous need for adjustment. The frequency of emergency missions was affected by uninfluenceable events as the COVID-19 pandemic. In Aachen’s EMS in March 2020, there were fewer non-urgent emergency missions than in a comparable period in 2018^39^. Other EMS systems have reported similar developments and suspected that patients were more reserved in calling for emergency assistance for medical requests^40^. For the tele-EMS system, the proportion of ambulatory treatments without needing hospital transport increased and unnecessary transports to hospitals were prevented by the tele-EMS system^39^. Especially the high rate of ambulatory contacts of 32.8% in in tele-EMS missions 2021 probably reflects the intention to save clinical resources during the pandemic. Nevertheless the dimension of the pandemic as a confounder is only evaluable to certain extend, as it was not possible in this study to analyze every single ambulance mission without the need for teleconsultation. This was not manageable in Aachen, as ambulances still document in hand-written mission protocols. The decline of tele-EMS missions from 2018 to 2019 due to the TEMS-study has already been elaborated previously in the discussion^18,26^.

To measure the outcome quality of structural changes in an EMS, it is essential that prehospital emergency care studies investigate the clinical outcomes of emergency patients. The missing link between pre-hospital and in-hospital data in EMS studies remains one of the biggest challenges in investigating novel developments in EMS.

## Conclusion

In summary, in our study investigating the long-term effects of a tele-EMS system, we found that onsite EMS physician incidence decreased and remote telemedical support for paramedics increased. Tracer diagnoses were the primary reasons for contacting tele-EMS physicians. The capability of simultaneous teleconsultations, as well as the reduction in call duration and increase in ambulatory treatments, demonstrate the increasing experience of paramedics and tele-EMS physicians with the system in place. A prehospital tele-EMS system represents an important component for mitigating the challenges faced by the prehospital health sector in Germany.

### Supplementary Information

Supplementary Table 1.

## Data Availability

The datasets analyzed in the current study are not publicly available because they are municipal property and cannot be published online under open-access agreements. However, these datasets are available upon reasonable request and with permission from municipal authorities. Correspondence and requests for materials should be addressed to H.S.

## Abbreviations

ACS: Acute coronary syndrome
COPD: Chronic obstructive lung disease
ECG: Electrocardiogram
EMS: Emergency medical service
Onsite EMS physicians: Onsite emergency physicians
SOP: Standard operating procedure
Tele-EMS physicians: Telemedical emergency physicians
Tele-EMS system: Telemedical systems for emergency medical services
