# Supplementary material for: Long-term effects of a prehospital telemedicine system on structural and process quality indicators of an emergency medical service
Source: Sci Rep. 2024 Jan 3;14:310. doi: 10.1038/s41598-023-50924-5 (PMC10764932; doi:10.1038/s41598-023-50924-5)
Supplement: Supplementary file 1 — Supplementary Table 1. [file 41598_2023_50924_MOESM1_ESM.pdf]

Supplementary file 1: Frequency of tracer (*) and non-tracer diagnoses from all tele-EMS-missions of the city of Aachen from 2015 to 2021 in descending order.

diagnoses	2015	2016	2017	2018	2019	2020	2021	total	%
analgesia total*	625	900	944	859	772	1260	1479	6839	31,1%
trauma extremity*	306	439	456	400	370	689	775	3435	15,6%
abdominal emergency*	236	354	359	359	286	408	532	2534	11,6%
lumbar pain*	83	107	129	100	116	163	172	870	4,0%
ACS*	219	225	266	391	359	434	482	2376	10,8%
stroke*	277	370	389	345	288	227	218	2114	9,6%
hypertensive emergency*	228	208	231	222	169	248	304	1610	7,3%
arrhythmia*	96	102	126	177	136	156	194	987	4,5%
syncope	87	126	122	120	93	129	285	962	4,4%
psychiatric emergency	75	83	75	82	53	210	217	795	3,6%
respiratory infection	50	56	60	52	46	247	203	714	3,2%
neurological emergency	70	92	81	72	82	91	105	593	2,7%
GI-infection	38	55	65	49	45	97	100	449	2,0%
seizure*	37	45	66	49	31	45	60	333	1,5%
sepsis*	19	54	51	73	50	63	13	323	1,5%
bronchial obstruction*	30	48	42	61	45	46	47	319	1,5%
other tracer*	92	121	242	138	129	67	14	803	3,7%
other non-tracer	340	335	526	306	270	377	618	2772	12,6%
**rescue missions**	2283	2820	3286	2996	2568	3697	4339	21989	100,0%

ACS = acute coronary syndrome, GI = gastro-intestinal, eCOPD= exacerbated chronic obstructive pulmonary disease. Other tracer = numbers include all further diagnoses with available SOP as presented in table 1. Other non-tracer = numbers include all further cases without available SOP as explained in Table 1 of the manuscript.
